# A conceptual methodological approach for addressing complex interdisciplinary challenges

**DOI:** 10.1098/rsos.252200

**Published:** 2026-10-07

**Authors:** Ruth M. Morgan, Emma Levin, Rowan Haslam, Christina Ballinger, Maurice Biriotti

**Affiliations:** ^1^ Arista Institute, UCL, London, UK; ^2^ Security and Crime Science, UCL, London, UK; ^3^ Centre for the Forensic Sciences, UCL, London, UK; ^4^ UCL Faculty of Arts and Humanities, UCL, London, UK; ^5^ Department of Psychiatry, University of Oxford, Oxford, UK

**Keywords:** interdisciplinary, policy, global challenges, research question, integrative novelty

## Abstract

Complex challenges are multi-faceted and, therefore, require the input of knowledge and expertise from different disciplines. However, the prevailing structures of academic institutions tend to be based on specialized disciplinary knowledge and expertise, rather than cross-disciplinary synthesis of the insights from disciplinary knowledge and expertise, reflexivity and holistic approaches. As such, the existing model for generating ‘excellence’ in novel and specialized research does not lend itself to the synthesis of existing disciplinary insight, where the long-term goals and short-term metrics of success are different. There is, therefore, a need to create opportunities for a new and complementary conceptual methodological approach to address persistent complex challenges. This paper proposes two tools designed to facilitate thinking about what types of disciplinary knowledge and expertise can be relevant to tackling complex challenges, and to identify what questions might spark productive and unconventional dialogues across disciplines that result in interdisciplinary insight. Approaching complex challenges using these tools addresses the need to deal with different facets of the challenge in concert, and the need for effective juxtaposition and combination of knowledge and expertise from multiple disciplines to develop interdisciplinary insight and approaches to craft questions and address the challenge.

## 1. Introduction

### 1.1. Complex challenges exist

There is a class of ‘challenge’ which is widely recognized to be ‘complex’ [1]. These challenges are characterized by urgency (a recognition that they are dynamic, developing at pace and that doing nothing to attempt to solve the challenge will allow it to develop), diffuseness (a sense that many geographically distributed stakeholders are both affected by and contributing towards the challenge) and systemic complexity (existing across many interlinked and interdependent processes and systems).

Climate change is one example of this type of complex challenge [2]. It is generally characterized as having many interlinked and interdependent processes and systems, both within the deeply integrated environmental systems of the biosphere, cryosphere, hydrosphere and atmosphere, and within the interlinked societal systems of individuals and collectives, state actors, supra-state actors, corporate actors and charitable actors influencing and influenced in turn by each other [3]. It is widely perceived to be a challenge that is urgent, particularly in the context of planetary boundaries and the potential for crossing even more of those critical thresholds within decades or years [4]. It is widely agreed that the challenge of climate change is characterized by geographical complexity, with uneven distributions of those contributing to the challenge and experiencing the symptoms of the challenge [5,6], in addition to complex geographical and geopolitical arrangements of corporate, charitable and political entities [7].

For these types of complex challenges, it is widely agreed that solutions or mitigations are required, but despite both the visibility and awareness of the challenge and the allocation of significant resources towards solving it, the challenge remains persistent and resistant to resolution [8]. For example, when considering climate change, it is clear that a significant amount of research and policy efforts have been dedicated to addressing this challenge, and yet the issues remain current with questions being raised about why three decades of climate mitigation may appear to have been ineffective [9].

Different disciplines have described the anatomy of such challenges in a range of different ways (e.g. [10–14]). Despite variations in disciplinary framings, vocabularies and the contexts within which these perspectives have been articulated, over the last 40 years, these complex challenges have been considered to be ‘wicked’ [15], ‘super wicked’ [14,16], or ‘practical’ [17] problems, ‘messes’ [18], ‘swamp’ [19]; ‘grand challenges’ [20], ‘soft’ systems [21] and ‘problem situations’ […] ‘that make the traditional mathematical modelling tools [associated with operational research]…ineffective’ [22, p. 731] (table 1).

**Table 1.** An illustration of recurring themes observed in examples of literature outlining the anatomy of complex problems.

			examples of literature outlining the anatomy of complex problems			
overarching theme		recurring themes	‘wicked problems’ (Rittel & Webber [15])	‘problem situations’ (Mingers [22])	‘super wicked problems’ (Levin *et al.* [16])	‘grand challenges’ (Ferraro *et al.* [20])
**definition and communication**	**1**	**challenging to articulate the problem**	‘There is no definitive formulation of a wicked problem’ (p. 161)	‘The problem itself is not well-defined with agreed objectives’ (p. 731)		
	**2**	**challenging to identify a success state for the problem**	e.g. ‘Wicked problems have no stopping rule (i.e. clear completion state)’ (p. 162)	‘‘Success’ requires the generation of a degree of agreement among parties involved’ (p. 731)		‘Seemingly intractable’ (p. 365)
**the integration of different perspectives**	**3**	**challenging to align and navigate multiple stakeholders (individuals and organizations)**		‘The process is primarily one of learning and negotiation rather than the technical solution of a problem’ (p. 731)	e.g. ‘The central authority needed to address [the problem] is weak or non-existent’ (p. 123)	‘their impacts extend beyond the boundaries of a single organization or community’ (p. 365)
	**4**	**requires integration of different perspectives (disciplinary and organizational)**	e.g. ‘The choice of explanation determines the nature of the problem's resolution.’ (p. 166)	‘The situations all involve several interested parties [that…] generally hold different perspectives about the problem situation’ (p. 731)		‘The problems cut across jurisdictional boundaries, implicate multiple criteria of worth’ (p. 365)
**the anatomy of the challenge**	**5**	**exhibits complexity and inter-connectedness**	‘Every wicked problem can be considered to be a symptom of another problem’ (p. 165)			‘characterized by many interactions and associations, and nonlinear dynamics’ (p. 365)
	**6**	**exhibits urgency and represents high stakes**	e.g. ‘Every solution to a wicked problem is a ‘one-shot’ operation; because there is no opportunity to learn by trial-and-error, every attempt counts significantly’ (p. 163)		‘Time is running out’ (p. 123)	‘Significantly and adversely affect human welfare and wellbeing’ (p. 365)
**uncertainty**	**7**	**exhibits high levels of uncertainty/unknowns**		‘There are many uncertainties and often a lack of reliable (or indeed any) data’(p. 731)		‘Uncertain - The problems and their evolution are difficult to forecast for the actors’ (p. 365)

Indeed, it is possible to identify seven recurring themes that emerge from the range of approaches to considering the anatomy of such challenges:

It is challenging to:

(i) articulate the challenge [15,22];
(ii) identify a success state for the challenge [15,22]; and
(iii) navigate and align multiple stakeholders (individuals and organizations) who have different perspectives on the challenge [16,20,22].

In addition, these challenges:

(i) require the integration of different perspectives (both disciplinary and organizational) [15,20,22];
(ii) exhibit complexity and inter-connectedness [15,20];
(iii) exhibit urgency and represent high stakes [15,16,20]; and
(iv) incorporate high levels of uncertainty and unknowns [20,22].

It is therefore possible to suggest that one of the most salient points identified repeatedly in these descriptions of complex problems [i.e. 15,16,20,22], and one possible reason for the lack of satisfactory solutions being identified when tackling complex challenges, is that such challenges cross multiple interconnected systems [20] and therefore require the integration of different individual and organizational perspectives [15,20,22], which may include different understandings of what might constitute a solution (e.g. [15,20,22]). Understandings of such challenges may also be informed by different forms of knowledge and expertise (e.g. [20,22]) (taken here to be the high-level, specialized domain knowledge or skills that have been acquired through training, study and practice that often allows intuitive and rapid problem solving and lateral thinking [23–25]).

### 1.2. Complex challenges require insight from multiple disciplines

One discourse that is particularly prominent in the published literature addressing complex challenges is that these challenges are characterized by having many intertwined dimensions [20] that span different disciplines and areas of institutional knowledge. As a result, historically, specific aspects of these challenges have tended to be addressed by single disciplines [26]. For example, the financial impacts of climate change have tended to be studied by economists and business forecasters (with certain disciplinary assumptions and conventions) [27], while variations in atmospheric circulation have tended to have been studied by atmospheric scientists, meteorologists and physicists (with different sets of assumptions and conventions) [28]. Given the many intertwined dimensions of complex challenges, it is increasingly acknowledged that single disciplines and forms of expertise are not able, in isolation, to meaningfully untangle and address all of the dimensions of the challenge [29]. To devize solutions, it is necessary to find ways to harness the insights of the many different (and disparate) forms of disciplinary knowledge and expertise simultaneously to address the multiple dimensions of the challenge coherently.

There is a sizeable and vibrant corpus of existing work that looks at the integration of different disciplinary approaches, with many works providing definitions of, and distinctions between, ‘multi-disciplinary’, ‘interdisciplinary’ and ‘transdisciplinary’ work (e.g. [30–32]). Within this discourse, there is a recurring theme that the ‘integration’ of different forms of disciplinary knowledge and expertise is necessary to address complex and urgent research questions (e.g. [33–35]), yet there are practical and systemic barriers to such interdisciplinary work (e.g. [33,34,36,37]). Commonly cited obstacles to the integration of multiple forms of disciplinary knowledge and expertise include a perception of lower success rates in securing funding for such projects [38], language barriers creating friction in communication [39], incompatible expectations and conventions around the process of publication [40], tensions between the methodological and philosophical assumptions of different disciplines [41], a need to navigate epistemological incompatibilities [34] and a lack of faculty positions for interdisciplinary researchers [42], with interdisciplinary researchers needing to navigate career progression pathways and institutional structures that assume disciplinary boundaries [35].

Despite these well-documented obstacles to conducting research that integrates multiple disciplinary forms of knowledge and expertise, some universities now offer taught courses under the label of ‘interdisciplinary studies’ (e.g. [43]), and some textbooks cater to undergraduate readers wishing to familiarize themselves with interdisciplinary theory and methods (e.g. [44,45]).

Accordingly, this paper acknowledges this context of being situated to some extent within a discourse that: (i) debates definitions and taxonomies of work that integrates knowledge and expertise informed by multiple disciplinary positions; (ii) articulates the necessity of such integration to explore complex, urgent or ‘wicked’ problems; and (iii) provides extensive discussion of the potential barriers or obstacles to such work. At the same time, this paper is not seeking to address the current debates about what interdisciplinarity is understood to be. Rather, it is acknowledged that to untangle complex challenges and articulate questions that address the roots of the challenge, it is necessary to adopt a form of disciplinary integration which is based on and derived from deep disciplinarity and institutional knowledge and expertise. Notably, in this paper, this form of disciplinary integration is premised upon the existence of distinctive disciplinary identities, forms of knowledge and expertise, workflows and assumptions, which generate specific types of insight that can then be transformed into holistic (and in some cases interdisciplinary) insight. As such, this paper uses the term interdisciplinary to capture the integration of knowledge from different disciplines and sectors to address a research problem (and similarly, Palmer *et al.* [46] do not distinguish it from transdisciplinary, which has been used to describe more integrative and transformational integrative approaches). Specifically, it seeks to focus on how insights from knowledge and expertise of multiple disciplines can be brought together and integrated in ways that can address root issues and generate novel questions and insights.

### 1.3. The prevailing model of institutions founded on disciplinary specialization

In many of the dominant models of knowledge-generation, it is possible to argue that research is valued by funding bodies [47], and peer-reviewed journals [48], when it displays novelty and/or innovation (often considered to be disruptive and generating new knowledge rather than a new combination of existing ideas). Disciplinary specialization is also expected and rewarded, with the positioning of research within a discipline and sub-discipline often seen as a prerequisite of demonstrating intellectual legitimacy [49]. Both of these trends can be viewed as well-intentioned (attempting to ensure that funded and published research is seen as worthwhile (novel)) and legitimate (situated within an existing intellectual tradition) [50,51]. However, these trends towards the utility and desirability of research being evaluated by its (more narrowly defined) novelty and disciplinary positioning have consequences when it comes to addressing complex challenges.

#### 1.3.1. The consequences of disciplinary specialization

The tendency to value disciplinary specialization has contributed to a fragmentary or siloing effect, with publications and activities separated along disciplinary lines [52], and increasingly the challenge of communication between sub-specialisms, leading to a lack of sharing of relevant methods or insights derived from disciplinary knowledge and expertise (e.g. [42]). This may be compounded by the use of specialist disciplinary vocabulary; most publications are written using vocabulary that has resonance within a specific field, using specialized terminology to demonstrate knowledge and expertise, and communicate complex concepts with precision [53]. However, this form of writing can also be seen as ‘jargon’ that is impenetrable to the general reader, and sometimes even those from other specialisms [54] by making information more difficult to absorb even if definitions are provided [54,55]. The valorization of specialization also leads to a situation where there is a career-scale expectation that researchers will stay within one discipline because not doing so can reduce productivity and penalize careers [56,57] and thus disincentivize work that attempts to combine or juxtapose insights from multiple disciplines. Therefore, the importance of translation becomes clear, not only the translation within sub-specialisms within a discipline, and the translation necessary between disciplines, but also translation that makes insight accessible to audiences outside a discipline.

#### 1.3.2. The consequences of valuing novelty

Findings that have a ‘disruptive’ influence, which prompt movement away from previous ideas, are generally considered to be novel [38]. Yet, it is also possible to consider that novelty can also come from findings that integrate existing ideas and have a ‘consolidating’ influence that directs attention back to previous insights [39]. Novelty that has a disruptive influence tends to be the form of novelty that is predominantly valued, which can lead to a number of consequences. If the valued metric of productivity for professional academics is ‘novel’ research or innovation [40], with the output medium of the peer-reviewed journal article recognized and respected as the ‘standard unit’ of academic activity [41,42] or an industry adopted application, work which is not deemed novel (or indeed finds negative results) and that does not lead rapidly to ‘demonstrably novel’ peer-reviewed journal articles or products in industry is disincentivized [40]. This means that revisiting and juxtaposing existing insights derived from disciplinary knowledge and expertise (consolidating novelty) tends not to be incentivized (as it is not, by some definitions, novel), and also tends to take more time, as has been seen with the ‘production penalties’ experienced by interdisciplinary researchers and research teams [57].

### 1.4. A new conceptual methodological approach

In the context of the prevailing model of university research founded on disciplinary specialization that entrenches disciplinary siloes and valorizes a specific form of ‘novelty’, it is possible to suggest that a different conceptual approach is necessary for work that specifically attempts to untangle the intertwined dimensions of complex challenges [29]. Easton [58] succinctly expressed this paradox at the heart of modern academia: ‘To understand the world it has seemed necessary to analyse it by breaking it into many pieces (i.e. the disciplines and their own divisions). But to act in the world, to try to address the issues for the understanding of which highly specialized knowledge was presumably sought, we need somehow to reassemble all the pieces’ [58, p. 12].

Accordingly, if there is a complex challenge that requires the reassembling of the different strands of relevant knowledge [58] in order to facilitate different disciplines to share their insights on the different facets of the complex challenge, then there is a need to:

(i) identify where the relevant but isolated knowledge and expertise lie, and the configurations in which these pockets of knowledge and expertise can be productively ‘hotwired’ together to create insight-generating questions and exchanges. In other words, to establish which bits of the disciplinarily fragmented academic system are addressing facets of the same challenge, but in siloes, and to establish the extent of relevant existing research, which could inform productive dialogue across disciplines; and
(ii) ‘translate’ between the different disciplinary languages, assumptions, and insights from knowledge and expertise (i.e. to formulate new insight-generating questions which are legible to users of different disciplinary vocabularies, concepts and methods and enable exchange).

These approaches go against the current expectations and paradigms of legitimate and valuable academic research. That is to say, part (i) involves activity that does not conform to the expectation of disciplinary specialization, while part (ii) involves retrospection and juxtaposition rather than ‘disruptive’ novelty [39].

## 2. A conceptual methodological approach for facilitating insight from knowledge and expertise from multiple disciplines to address root issues of challenges and generate novel questions and insights.

### 2.1. The need for a new complementary conceptual approach that enables research that can achieve disciplinary integration and (interdisciplinary) insight

The current systems in research institutions tend to reward the specialization of disciplinary knowledge and expertise and novelty of research, and as a result, there are a number of paradigms, tools and frameworks which act to facilitate ‘novel’ projects undertaken by specialized researchers. In order to disentangle the dimensions of complex challenges, by effectively harnessing different forms of knowledge and expertise concerning specific dimensions of the complex challenges, it is argued that a different, complementary conceptual methodological approach is required. This approach needs to acknowledge that the long-term goals and short-term metrics of success are different for forms of research that address complex challenges and achieve novel (and sometimes interdisciplinary) insights and solutions. For example, it has been documented that interdisciplinary researchers and research teams may appear to have lower productivity [56,57], so longer periods of evaluation may be appropriate.

### 2.2. The consideration of a new conceptual methodological approach

Within the field of ‘systems thinking’, there is a diverse array of tools designed to assist with the visualization, contextualization and comprehension of complex systems (e.g. [59–61]). Such tools include ‘rich pictures’ providing opportunities to better understand a system (e.g. [60–62]); ‘pig models’ designed to assist exploration of various perspectives between different stakeholders [61]; and ‘casual loop’ diagrams designed to help visualize the casual interactions between different parts of the system, identify where feedback loops exist, and locate where in the system interventions might be effective [59–61]. There is also a rich diversity of tools associated with ‘design thinking’ (i.e. ‘thinking like a designer’ [63,64]), which can also be applied to complex challenges [65]. Like the ‘systems thinking’ tools mentioned above, these ‘design thinking’ tools, such as ‘system maps’ and ‘service blueprints’, can help to visualize and comprehend complex systems and the interactions of various elements of those systems [65].

Unlike tools that have been presented previously in the fields of design thinking and systems thinking which tend to address practical challenges (such as the roll out of electric vehicles [61]), the tools in this paper are explicitly targeted at, and intended to assist with, the practice of developing questions that enable interdisciplinary research. This paper presents a conceptual methodological approach comprised two tools designed to facilitate a complementary approach that enables productive integration of knowledge and expertise from different disciplines that address complex challenges and lead to interdisciplinary insights and solutions. The tools presented here are not seeking to visualize and identify feedback loops (as in the casual loop diagrams) or to fully capture the gamut of stakeholder perspectives (as in pig models). Rather, the tools are designed to facilitate thinking about what types of disciplinary knowledge and expertise can be relevant in addressing a complex challenge, and to identify what questions might spark productive and unconventional cross-disciplinary conversations that result in interdisciplinary insight.

The form of the tools evolved from a series of discussions between a group of disciplinary researchers from the sciences, social sciences, humanities and engineering. Each researcher brought experience of working across disciplines, and the discussions drew upon the disciplinary knowledge and expertise each researcher brought to the discussion, which was modelled on the format of the modern renaissance workshop [66] in an open and ongoing manner. Findings were synthesized to identify broad themes of synergy, as well as gaps and tensions (which were often mutually incompatible). The dominant themes that emerged were then scrutinized to identify generalizable themes and framing.

## 3. Two tools: framing the challenge and generating new questions

The first tool, the ‘framing wheel’ (figure 1) has been designed to aid thinking about what types of disciplinary knowledge and expertise may be relevant to a specific complex challenge. The second tool, the ‘question generator’ (figure 2) has been designed to generate questions that may spark productive dialogue and interactions between disciplines with a goal of exploring possible solutions to a complex challenge.

**Figure 1.**
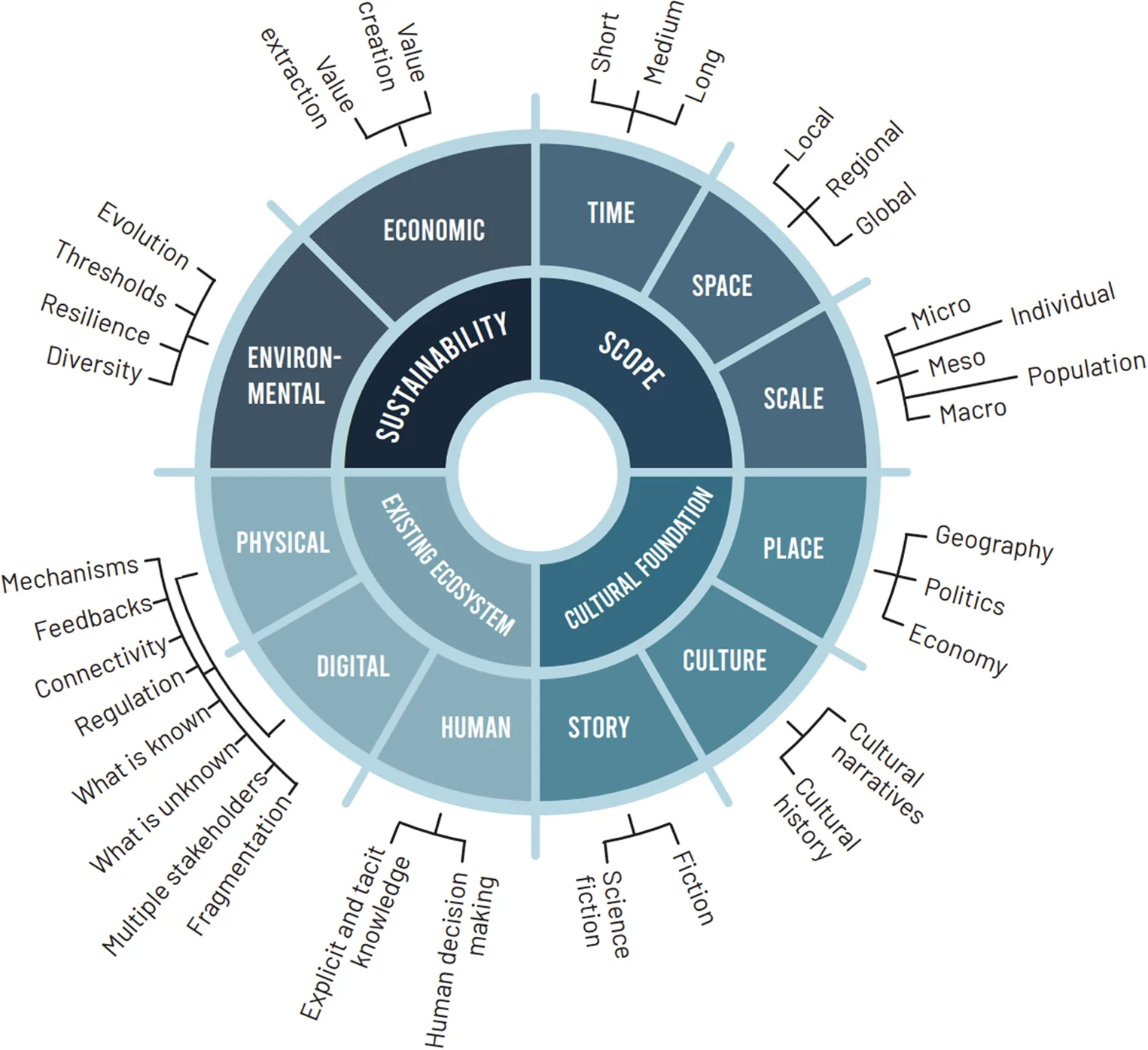
Tool 1: the systems-level framing wheel.

**Figure 2.**
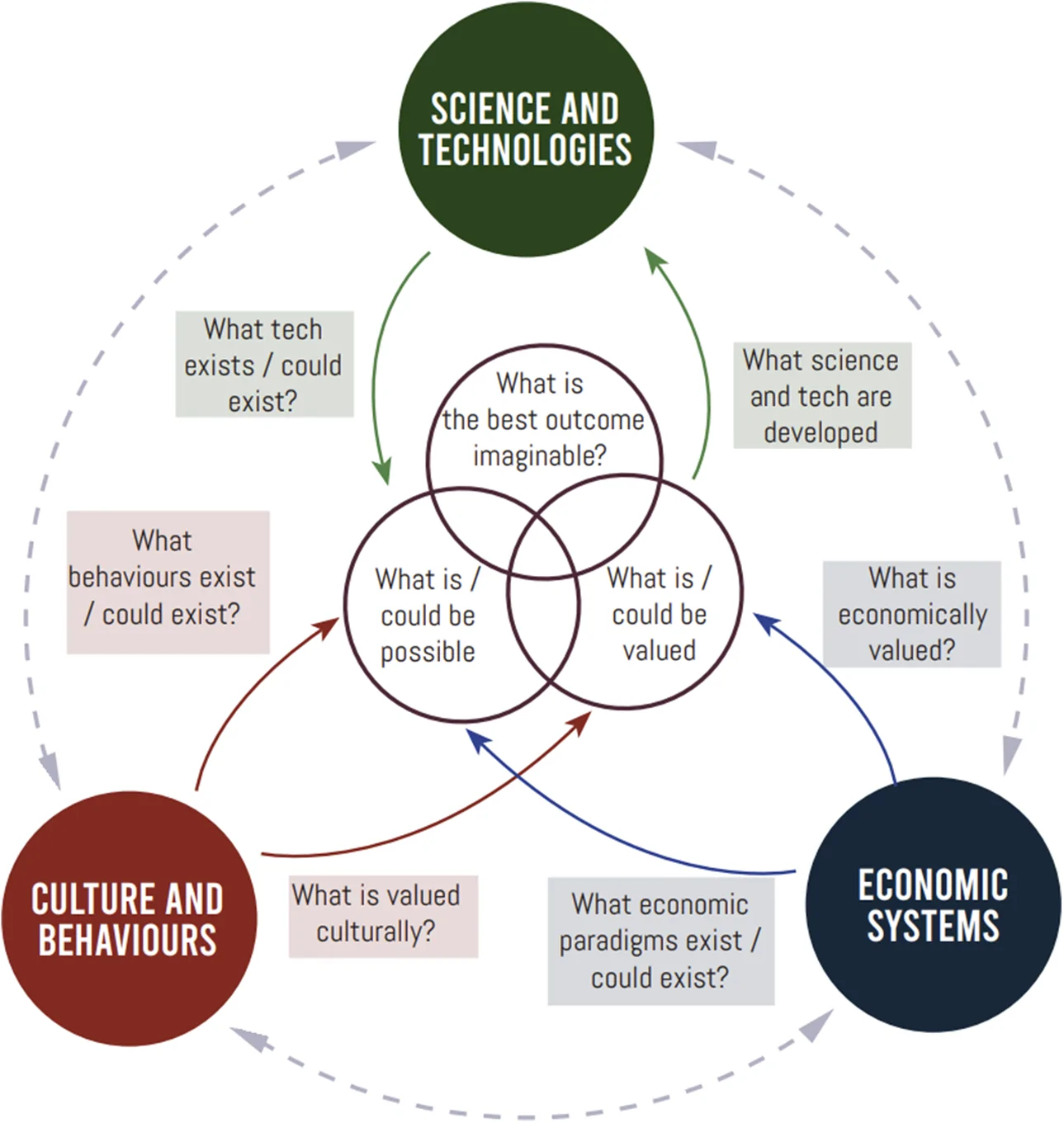
Tool 2: a question generating tool.

### 3.1. Tool 1: a systems-level framing wheel

In order to establish what types of disciplinary knowledge and expertise may be relevant to a complex challenge, describing the anatomy of the challenge is an important step. This in itself may be a difficult task when a complex challenge is defined by the difficulty of identifying all of the intermeshed, interlinked and interactive dimensions [20]. Figure 1 presents the first tool designed to prompt consideration of the dimensions of a specific complex challenge, and the disciplinary knowledge and expertise that may offer insights relevant to addressing them. The wheel is structured in four parts or quadrants that emerged from the range of discussions in the workshops, to assist a consideration of the scope of the complex challenge, its cultural foundations, the existing ecosystems that surround it and how the challenge intersects with questions of environmental and economic sustainability (table 2). These are not mutually exclusive categories, and are necessarily simplifications. Some important aspects are not represented explicitly (such as justice, for example, which is incorporated implicitly in the consideration of the cultural foundation and sustainability quadrants), but the aim is that the tool offers a starting point with helpful prompts to consider the challenge from a broad range of perspectives.

**Table 2.** Using tool 1 (the systems-level framing wheel) to map and explore the anatomy of a complex challenge. It is anticipated that researchers can make use of this tool in the early scoping of a research project to explore the dimensions of a particular challenge and identify key dimensions of the challenge that inform the generation of the research question(s) (using tool 2 (figure 2)).

quadrants of the framing wheel	considering	example from an exemplar complex challenge of climate change.
**scope**	what is the temporal and spatial extent of the complex challenge? at what scales are processes operating, benefits or harms manifesting, and key stakeholders engaging?	is the challenge to be addressed within a political time frame (2–5 years), anthropogenic time frame (10–50 years) and geomorphological/geological time frame (100–1000 years)? is the key issue driven by plate tectonics, orbital variations (Milankovitch cycles) or biochemical cycles (various scales)?
**cultural foundation**	what insights do intellectual traditions that have an awareness of place, space and culture contribute? E.g. what do geographers, economists, anthropologists, historians and those who study political systems identify? Culturally, what narratives and narrative histories abound? what can be gleaned from stories told about the challenge, in (science) fiction or journalism?	what different forms of knowledge address climate and climate change? what are the stories being told about climate change (addressing the role of human agency and impact, the role of thresholds and planetary boundaries etc.)? whose stories are they?
**existing ecosystems**	who are the actors participating in and affected by the challenge, and what are the processes, mechanisms and feedback loops? What is currently regulated, and what are recognized as regions of uncertainty or the unknown? If the system is heavily fragmented, what appear to be the lines of fragmentation, and where do connections exist?	who are the international, national, regional and local actors? what are the established physical mechanisms and feedback loops of climate change and where is there contest? what is happening in the digital ecosystem that is contributing to the challenge of climate change? what are the different societal (human) scenarios that have similar and different experiences of climate change? what are the international, national, regional and local regulations and legislations in force? where is uncertainty acknowledged in the physical ecosystem, the digital ecosystem and human ecosystems?
**sustainability**	how is economic value created and extracted in the challenge domain? is the challenge likely to affect resilient or fragile ecosystems? could the challenge affect a planetary threshold [4]? do the environmental concepts of thresholds, resilience and diversity relate to the challenge?	what are the economic considerations of climate change at different scales? what contexts are considered to be resilient or fragile to climate change (over what time frame and spatial scale)? is the issue of enquiry when considering climate change deemed to be a question of increasing resilience, or minimizing harm?

### 3.2. Tool 2: a question generator

As discussed in §1, many complex challenges exhibit dimensions that intersect and interact. Many complex challenges tend to exhibit three specific dimensions: (i) a technological or scientific dimension, alongside (ii) a cultural and behavioural dimension (which incorporates societal context, institutional context, legal and regulatory context, and ethical considerations), alongside (iii) an economic dimension (which incorporates the political context and governance structures). To return to climate change as an exemplar, this can be observed in the form of: (i) a technical dimension of what technologies of energy generation exist, and what technologies of environmental repair exist, alongside (ii) a cultural and behavioural dimension of what cultures and behaviours of energy consumption and environmental repair exist, and (iii) an economic dimension of what economic systems and paradigms exist. It may be possible to suggest that these three dimensions tend to interact with each other in multiple ways, and that the interaction of all three contributes to the context of the complex challenge, informing what is seen to be valued, what is seen to be possible and what is seen to be optimal (figure 2).

The interaction of the technologies, cultural behaviours and economic systems that currently exist or are perceived to plausibly exist in the near future, informs what intervention to a complex challenge is seen as currently possible. To return to the challenge of climate change, one technical dimension of the challenge is that many nations currently rely on technologies designed to run on fossil fuels (e.g. power stations, and vehicles using internal combustion engines) [67]. A cultural dimension includes the perception that many people do not want to or expect to radically alter their ‘normal’ patterns of consumption, or activities, in order to tackle climate change [68]. An economic dimension could be considered to be the prevailing global economic paradigm (late-stage neoliberalism) that values growth and would view a decline in growth as unacceptable [69]. Accordingly, the three dimensions combine to suggest that an intervention which immediately prohibited the use of existing fossil fuel technologies would not be seen as economically, culturally or technologically possible.

Similarly, existing behaviours, cultural traits and economic systems together inform what is seen as currently valued, or what could plausibly be valued. This allocation of value then determines what science and technologies are developed. For the challenge of climate change, amongst some groups of the population there is a cultural desire to avoid or repair environmental damage. Economically, there is a situation where energy provision is organized at the scale of nation-states, and where many governments are keen to avoid dependence on international oil and gas imports (and therefore vulnerability to volatile international oil and gas prices). This results in a situation where cheaper and greener energy sources are valued, and the technologies of solar and wind power receive both industrial attention and nation-based subsidies.

The best outcome imaginable is a function of what is seen as valued (best) and possible (imaginable). In the climate change example, the best imaginable outcome might be seen as a liveable planet (culturally valued) with continued economic growth (economically valued), which does not involve huge cultural disruption or fundamental reinvention of economic paradigms (which would be unimaginable). Accordingly, where a complex challenge seems intractable, exploring the three dimensions and how they interact can precipitate the generation of questions which cut across traditional disciplinary remits. For example, in the climate change challenge domain, useful questions to consider might be: what new technologies could change what is considered possible? and what interventions could change the cultural and economic dimensions to promote the development of those new technologies? For example: some renewable energy types have enormous potential for energy generation, but do not operate at a uniform rate, making energy storage an underlying technical challenge [70], as with solar power, where energy generation is restricted to daylight hours, and where weather conditions and seasonal fluctuations create variance in output over time [70]. One possible question, which would touch upon technical, cultural and economic dimensions of the challenge would be: ‘what could change to incentivize the development and implementation of energy storage systems for renewable energy sources with intermittent outputs?’

Therefore, tool 2 as a ‘question generator’ (figure 2) might be helpful in assisting the identification of components of a sub-optimal system which could be changed (table 3); and if a challenge appears intractable, explore what might make it tractable.

**Table 3.** Using tool 2 (a question generator) to identify components of a system that could be changed or move from intractable to tractable and assist in the crafting of research question(s).

elements of the ‘question generator’	description
**science and technologies**	current scientific and technological capabilities shape culture and behaviours and economic systems. Conversely, scientific and technological capabilities are shaped by current culture and behaviours, and by current economic systems. What is perceived as ‘valued’ shapes, what science and technologies are developed and what is seen as possible is a function of what technologies or scientific breakthroughs either exist or could plausibly exist. Derived questions will likely address: • what technologies currently exist or could exist? • what scientific capabilities currently or could exist?
**culture and behaviours**	current culture and behaviours exert influence over scientific and technological capabilities and economic systems. Equally, the current culture and behaviours are shaped by current economic systems, and by the technologies and scientific capabilities which are currently available. What is perceived as culturally ‘valued’ shapes, what is seen as the best outcome imaginable and what is seen as possible is a function of what behaviours either exist or could plausibly exist. Derived questions will likely address: • what behaviours or cultural contexts currently or could exist? • what is or could be valued culturally?
**economic systems**	current economic systems exert influence over scientific and technological capabilities and culture and behaviours. Equally, the current economic systems are shaped by culture and behaviours, and by currently available technologies and scientific capabilities. What is perceived as economically ‘valued’ shapes, what is seen as the best outcome imaginable and what is seen as possible is a function of what economic paradigms either exist or could plausibly exist. Derived questions will likely address: • what economic systems currently or could exist? • what is or could be valued economically?
**outcomes and values**	the (broadly defined) best outcome imaginable is a function of what is seen as possible and what is seen as valuable. Economic systems and culture/behaviours may shape what is perceived as valuable. Changes to culture/behaviours, economic systems or the capabilities of science and technologies may change what is seen as possible. Derived questions will likely address: • what is or could be seen as possible, valued or optimal?

## 4. Discussion

### 4.1. The value of exploring complex challenges from insights derived from different knowledge and expertise from multiple disciplines to identify root causes

Tool 1 offers an approach to exploring the anatomy of a complex challenge. Posing a series of questions (figure 1) that consider different attributes of the challenge at a range of scales (spatial and temporal) and contexts opens up opportunities to articulate the anatomy of the challenge from different perspectives and identifies core issues. Tool 2 then offers one approach to tackling the key domains of a challenge area (science and technologies, culture and behaviours, economic systems (figure 2)) simultaneously in a collective manner.

### 4.2. Interconnectedness as a fundamental quality of complex challenges

The interconnectedness of different dimensions of complex challenges means that ‘fixing’ one element of the challenge in isolation does not usually result in an overarching solution that addresses the whole challenge and may indeed have unintended consequences or knock-on effects. At this level of simplification, to address a complex challenge, it is necessary to attempt to solve every aspect of the challenge simultaneously and in concert. In general, and with respect to the three dimensions outlined in figure 2, addressing policy and funding in isolation does not usually result in a solution to the whole challenge without also addressing new technologies or societal values and consent [71]. Similarly, developing new technologies to address a challenge without addressing policy/funding, societal values and acceptance does not usually result in effective solutions [72]. Indeed, establishing societal acceptance will not usually change a situation if the technologies needed do not yet exist (and/or are not enabled/funded). Of course, in practice, it is rare to be able to achieve such a holistic approach. It is important to acknowledge that helpful progress can be achieved by addressing some aspects, particularly if it is possible to identify where co-benefits may lie (for example, the value of solving one aspect of a challenge may be increased if another aspect is considered at the same time).

### 4.3. The need to navigate nested cascades of issues and symptoms

In complex challenges, there is a tendency for issues to cascade and create further challenges. For example, with a warming trend, there is greater ice melt from glaciers, which can lower albedo, creating a feedback loop of warming [73]. Similarly, warming trends can increase the geographical ranges for insects which spread zoonotic disease (e.g. malaria/*Anopheles* mosquitoes, or Lyme disease and ticks), causing health challenges [74].

When issues cascade and compound, it is necessary to investigate the relationships between the issues, and to identify the source of the challenge or so-called ‘root problem’ to address those that cascade outwards. It may be initially unclear which are the symptoms and which are the causes. It may be that in the short term, the symptoms appear easier to address. However, ultimately, addressing symptoms will not effectively address the challenge of identifying holistic solutions.

### 4.4. The importance of insight derived from deep disciplinary knowledge and expertise for deriving synthesized interdisciplinary insights that can drive holistic solutions

Complex challenges require insights derived from knowledge and expertise from multiple disciplines to capture the range of interconnected issues and attributes of the challenge (figure 1). To explore holistic solutions that address society (culture and behaviour), policy (economic systems) and science and technologies, and insights from the knowledge and expertise from multiple disciplines in concert, there is a need for synthesis of the disciplinary insights derived from disciplinary knowledge and expertise (figure 2). As such, the two tools presented in this paper aim to offer an approach to achieving this synthesis as a means for considering potential solutions to complex challenges that address the root causes in ways that address the whole challenge.

### 4.5. Limitations and future directions

The conceptual methodological approach presented here is designed to complement existing approaches that have been articulated in the systems-thinking, design-thinking and broader literatures that address the importance of holistic approaches to engaging with complex challenges. Each tool presented here necessarily represents a simplification that is designed to equip researchers with an initial approach with which to bring multiple forms of knowledge and expertise to bear to address complex challenges in ways that may result in interdisciplinary insight. As such, each tool clearly has limitations and, if used inappropriately, could oversimplify the complexity being considered. The conceptual methodological approach is not designed to act as a checklist or a process that could be automated without appropriate nuance. Rather, it is hoped that the two tools offer a conceptual methodological approach that is flexible and useful in terms of identifying important attributes (tool 1), highlighting where resonance and tensions may lie and where translation may be needed. This can then pave the way for developing new questions (tool 2) that can lead to a synthesis of knowledge and expertise from multiple disciplines, which paves the way to interdisciplinary insight that can unlock seemingly intractable challenges.

In the future, it is anticipated that the use of this conceptual methodological approach will result in iteration and refinement as the range of complex challenges considered grows. The weighting of different attributes considered in tool 1 will need to be considered on a case-by-case basis, along with how value can be articulated in different scenarios when using tool 2. Worked examples will offer additional insights to assist the use of the tools and approach.

## 5. Conclusion

Complex challenges have many attributes which require insights derived from the knowledge and expertise from multiple disciplines to identify root causes. To attempt to solve these complex challenges, it is therefore necessary to engage and integrate the insights derived from the knowledge and expertise of different disciplines which are often not in direct dialogue and interaction, or in contexts where effective translation is feasible. In order to enable effective and direct interaction between different forms of disciplinary knowledge and expertise, it is necessary to: (i) identify the relevant dimensions of disciplinary capability, knowledge and expertise; (ii) translate between different disciplinary languages, assumptions and framings within each disciplinary discourse; (iii) identify fruitful questions; and (iv) synthesize the different insights derived from disciplinary knowledge and expertise, creating a metaphorical intellectual short circuit that leads to interdisciplinary approach(es). Traditionally, this has been difficult, because it is rare that there is an explicit connection between discourse that spans different disciplines and impact on real-world challenges, because dialogue across disciplines and its associated synthesis takes time [56,57], institutions have intrinsic and deep disciplinary siloes, and excellence is recognized and rewarded within disciplinary parameters.

The two tools presented in this paper offer a conceptual methodological approach that seeks to address complex challenges and achieve rigorous interdisciplinarity to tackle challenges holistically and find solutions. The first tool is designed to allow researchers to explore the anatomy of a complex challenge and identify key dimensions of a challenge (i). This approach creates the opportunity to identify resonance and tension between different dimensions of the challenge, and allows researchers to begin to engage with the different disciplinary languages, assumptions and framings that may exist within each dimension (ii). The second tool is designed to assist researchers in identifying components of the system in which those resonances and tensions reside, and consider where aspects of the intractability of the challenge may lie. Considering which components of the system could be reimagined or changed to move the challenge from seemingly intractable to being more tractable, then lays a foundation from which research question(s) can be crafted (iii). It is hoped that this conceptual methodological approach will then lay a foundation from which it will be possible to synthesize and derive interdisciplinary approaches that address core issue(s) and move a challenge from seemingly intractable to more tractable (iv).

Complex challenges are not going away. If anything, the opportunities for the growth of existing and development of new complex challenges are growing. It is therefore necessary to create space for different insights derived from the knowledge and expertise of multiple disciplines that can lead to interdisciplinary approaches that make it possible to tackle a whole challenge (every aspect of the challenge simultaneously) rather than addressing individual elements of the challenge in isolation.

## Data Availability

This article has no additional data.
